# The Moderating Effects of Young Adults’ Personality Traits on Social Media Immersion

**DOI:** 10.3389/fpsyg.2020.554106

**Published:** 2020-11-02

**Authors:** Tai-Kuei Yu, Neng-Huei Lee, Cheng-Min Chao

**Affiliations:** ^1^Department of Business Administration, National Quemoy University, Kinmen, Taiwan; ^2^Department of Business Administration, National Taichung University of Science and Technology, Taichung, Taiwan

**Keywords:** internet use, young adults, personality traits, social media, immersion

## Abstract

Young adults are currently among the heaviest users of Internet-based social media applications. The goal of this study was to develop and empirically validate a conceptual model to test associations between students’ attitudes toward social media and their experiences in social media use and immersion. Participants were 9,633 students (average age 16 years; 4,702 males, 4,931 female) who randomly selected from 150 high schools in Taiwan. Participants completed questionnaire surveys describing their attitudes toward social media, immersion experiences, and Big Five personality traits. Structural equation modeling was used to determine factors that predicted and moderated social media immersion. The results of this study highlight the impact that specific personality traits have on the connections between attitudes toward social media and the immersion young adults experience when engaged with social media platforms. These findings suggest that schools and families should establish guidelines to protect young adults from excessive immersion in social media usage, ensure the safety of online environments for this user group, and inform youth regarding the proper use of social media.

## Introduction

The Internet has spurred the development and widespread use of new forms of social media (Correa et al., 2010; Zhang et al., 2015; Hu et al., 2017; Manwong et al., 2018). Social media and multidimensional platforms allow users to exchange information and discuss ideas through posting, commenting, chatting, and other actions. Importantly, these platforms allow users to access knowledge and exchange information expeditiously, communicate easily and cost efficiently, and create collaborative environments (Zhang et al., 2015; Kaya and Bicen, 2016; Hu et al., 2017; Manwong et al., 2018; Yu et al., 2018). In addition to facilitating learning, communication, and collaboration, social media provides supportive leisure and entertainment environments (Kaya and Bicen, 2016; Zha et al., 2018).

Adolescents are currently among the heaviest users of Internet-based social media applications (Lu et al., 2016). Terzi et al. (2019) stated that social media is a broad field of internet, which encompasses social network sites (Facebook, Twitter, IG, etc.), cooperative websites (Wikipedia), professional networks (LinkedIn), gaming websites, and YouTube. These websites have accumulated huge, international bases of high school and young adult users (Terzi et al., 2019). Adolescents also use social media websites and tools to interact online, which enhances the dynamics of interpersonal relationships (Wang et al., 2012; Balakrishnan and Gan, 2016; Kaya and Bicen, 2016; Kaczmarek et al., 2017; Alomar et al., 2019). For example, Pokémon Go is a popular augmented reality game in which players can socialize face-to-face with other players or interact via social networking sites (Kaczmarek et al., 2017; Alomar et al., 2019) to build teams, socialize, exchange experiences or ideas, and develop friendships.

Csikszentmihalyi (1997) pointed out that “consumers may experience an immersive state of flow in a variety of activities” (Csikszentmihalyi, 1997; Hamilton et al., 2016). Witmer and Singer (1998) defined immersion as a “psychological state characterized by perceiving oneself to be enveloped by, included in, and interacting with an environment that provides a continuous stream of stimuli and experiences” (p. 227). Jennett et al. (2008) argue that immersion has three features: (1) lack of awareness of time, (2) loss of awareness of the real world, and (3) involvement and a sense of being in the task environment. According to Csikszentmihalyi’s (1997) perspective, this study argued that young adults may experience an immersive state of flow in the social media activities. In addition, based on the perspective of Jennett et al. (2008), this study also stated that excessive engagement with social media may negatively impact young adults. When students use social media for a long time and focus on the social media usage immersion may result, and three characteristics were occurring, include (1) lack of awareness of time, (2) loss of awareness of the real world, and (3) involvement and a sense of being in the task environment (Csikszentmihalyi, 1997; Jennett et al., 2008; Terzi et al., 2019).

In this study, we investigated the effects of attitudes toward social media in Taiwan young adults and the effect of these attitudes on immersion in social media. However, previous studies (e.g., Kim et al., 2013; Chen et al., 2015; Choi and Shin, 2017; Xiao and Mou, 2019) have demonstrated that influence of social media use on individuals’ cognitions, attitudes, or behaviors is different for different types of individuals. Personality traits (e.g., Big Five) could moderate the influence of social media use on attitude formation (Kim et al., 2013; Chen et al., 2015; Choi and Shin, 2017). Moreover, few researchers have addressed dispositional factors such as attitudes and personality traits, moderator effects, or predictors of the mental absorption experienced. Therefore, this study developed a conceptual model regarding the effects of students’ attitudes toward social media on immersion, with personality traits as moderating effects, then empirically tested it with structural equation modeling (SEM). This study aims to address the following research questions: (1) Do students’ attitudes toward social media influence their immersion experiences? (2) How do students’ personality traits (i.e., extraversion, agreeableness, openness to experience, conscientiousness, and neuroticism) moderate the effects of attitude toward social media on immersion? The results are expected to enhance understanding of social media immersion among adolescence, provide recommendations for schools and government education authorities, and elicit the effects of social media use on psychoeducation and related problems.

### Immersion in Social Media

Flow theory has been widely used to explore individuals’ attitudes, behaviors, and experiences in various contexts (Chen et al., 2017; Hu et al., 2017). In recent years, flow theory has been applied to information technology to explain human–computer interactions and to individuals’ use of social media and social gaming (Hu et al., 2017; Chen et al., 2017; Liu et al., 2018). Flow is usually characterized by concentration and focus, loss of self-consciousness, and loss of a sense of time (Csikszentmihalyi, 1997). Csikszentmihalyi (1997) noted that flow is experienced when individuals fully engage or immerse themselves in specific activities. Hu et al. (2017) defined flow as “the feeling of enjoyment and pleasure arising from deep immersion in an activity.” Cruz and Uresti (2017) pointed out that the flow state has several characterizations, including being less conscious of the passage of time, full immersion in the task, and feeling in complete control.

Flow and immersion are both psychological states and have many of the same characteristics (Cuny et al., 2015; Cruz and Uresti, 2017). Although immersion is associated with the concept of flow, immersion is a broader process. This study argued immersion results from individuals’ interaction with a social media environment and thus relies on the features of the social media usage. Experiencing complete, indulgent focus on the social media usage, free from distraction, is often referred to as immersion (Burns and Fairclough, 2015; Hamilton et al., 2016; Liu et al., 2018). Some previous studies (Jennett et al., 2008; Hamilton et al., 2016) suggest that immersion is beneficial in a variety of activities. Immersion in video games and virtual worlds are considered important for user enjoyment (Christou, 2014; Grinberg et al., 2014; Burns and Fairclough, 2015). Immersion refers to the experience that completely invades individuals’ perceptive and emotional systems and psychological processes, so that immersed individuals experience engagement, engrossment, and total immersion (Brown and Cairns, 2004; Cuny et al., 2015; Cruz and Uresti, 2017). Most research focuses on interaction with digital worlds, especially video game and virtual spaces (Christou, 2014; Burns and Fairclough, 2015). Social media not only provides virtual games environment but also provides a simple method for high school students to search for knowledge, share information, ask questions, and engage in leisure and entertainment. These activities have been shown to improve student interest and engagement in knowledge absorptive (Balakrishnan and Gan, 2016), as well as improve leisure and entertainment. When students use social media more frequently or for a long time, especially for leisure or entertainment, problems with immersion occur.

Although immersion has been an important topic regarding the study of virtual worlds (e.g., video games and research tools), few studies have covered the components which cause the phenomenological experience of immersion in virtual social media worlds, including the importance of the users’ attitude toward social media. According to previous researchers (Jennett et al., 2008; Cuny et al., 2015; Hamilton et al., 2016; Cruz and Uresti, 2017), this study defines immersion as a psychological state in which young adults are fully engrossed within the social media environment and focus on the social media usage, free from distraction. To our knowledge, there are no existing studies investigating the theoretical foundation of possible links between attitudes toward social media and immersion. Social media has been widely developed and used and has become an integral part of the lives of young adults. According to Csikszentmihalyi (1997), young adults may experience an immersive state of flow in the social media activities and focus on the social media usage, free from distraction. Therefore, this study argued that when young adults have well social media attitudes, they will experience an immersion in social media activities as better. Accordingly, in this study, we hypothesize that attitudes toward social media have a significant effect on immersion.

Hypothesis 1: Attitude toward social media has a significant effect on immersion.

### Personality Traits

Recognizing the potential importance of social media use to the development of adolescents and young adults, researchers have been working to identify the personal characteristics that best predict social media use (Wang et al., 2015; Marino et al., 2016; Azucar et al., 2018). Many previous studies have analyzed the effects of gender and various personality traits on social media use (Clemens et al., 2015; Chen et al., 2016; Azucar et al., 2018); findings indicate that some personality traits are associated with interpersonal interactions and social media use (Wang et al., 2015; Chen et al., 2016; Marino et al., 2016; Azucar et al., 2018). Several scholars have examined the influence of the “Big Five” personality traits on social media use. The popular Big Five model categorizes personality traits into five domains: agreeableness, openness, extraversion, neuroticism, and conscientiousness (McCrae and Costa, 2004; Wang et al., 2012; Chen et al., 2016; Tang et al., 2016; Choi and Shin, 2017).

The findings of Tang et al. (2016) suggest that certain personality traits, including agreeableness, openness, extraversion, neuroticism, and conscientiousness, are associated positively with the use of social media. Wang et al. (2012) argued that personality factors are related to an individuals’ use of social networking sites and found that extraversion, neuroticism, and openness played important roles in how social networking sites were used. Correa et al. (2010) found that extraversion and openness to experiences were positively related to social media use and emotional stability (high neuroticism) negatively predicted social media use. Choi and Shin (2017) analyzed the moderating effects of the Big Five personality traits in a homogeneous or heterogeneous community on the relationship between social media use and political compromise. The results found that agreeableness and conscientiousness moderated the influence of social media use on attitudes toward political compromise. However, the non-significant interaction among social media use and extraversion, emotional stability, openness. Xiao and Mou (2019) found that two of the Big Five personality traits, neuroticism and extraversion, moderates the impact of social media characteristics on stressors. In addition, Kim et al. (2013) found that extraversion and openness to experiences moderate the influence of social media on discussion network heterogeneity and civic participation. Chen et al. (2015) explored the moderating effects of personality traits on the relationship between the motive traits (need for affiliation and need for popularity), self-esteem traits (self-esteem) and self-disclosure on Facebook. The results demonstrate that (1) conscientiousness and emotional stability moderate the relationship between need for affiliation and self-disclosure; (2) openness to new experience, emotional stability, and extraversion moderate the relationship between need for popularity and self-disclosure; (3) agreeableness, conscientiousness, and extraversion moderate the relationship between self-esteem and self-disclosure.

According to the above discussion, most previous studies have examined personality traits as factors external to social media use and found that certain personality traits (i.e., extraversion, neuroticism, and openness to experiences) are important external factors that affect individuals’ use of social media. In addition, some social media studies (i.e., Kim et al., 2013; Chen et al., 2015; Choi and Shin, 2017; Xiao and Mou, 2019) also found that the Big Five personality traits have moderating effects. However, to our knowledge, no research has examined whether young adults’ individual personality traits (i.e., extraversion, agreeableness, openness to experience, neuroticism, and conscientiousness) moderate the relationships between the independent variables (attitude toward social media) and the dependent/outcome variable (immersion). Accordingly, we argued that extraversion, agreeableness, openness to experience, neuroticism, and conscientiousness moderate the impact of attitude toward social media on immersion. Specifically, the following hypotheses were proposed:

Hypothesis M1: The relationship between attitude toward social media and immersion is moderated by the level of extraversion.

Hypothesis M2: The relationship between attitude toward social media and immersion is moderated by the level of agreeableness.

Hypothesis M3: The relationship between attitude toward social media and immersion is moderated by the level of openness to experience.

Hypothesis M4: The relationship between attitude toward social media and immersion is moderated by the level of neuroticism.

Hypothesis M5: The relationship between attitude toward social media and immersion is moderated by the level of conscientiousness.

## Methodology

The empirical analysis uses data from a survey of 9633 high school students from both high schools and vocational high schools in Taiwan, aged between 15 and 20 years. SEM was used to analyze the causal effect of six hypothesized predicting factors (includes five moderating effect). SPSS 18.0 was used to analyze sample and describe statistics, while for the analysis of causal relationships, moderating effect, and hypotheses testing, PLS 30 was applied for parameter estimation and structural equation model (“SEM”) evaluation.

### Research Model

**FIGURE 1:**
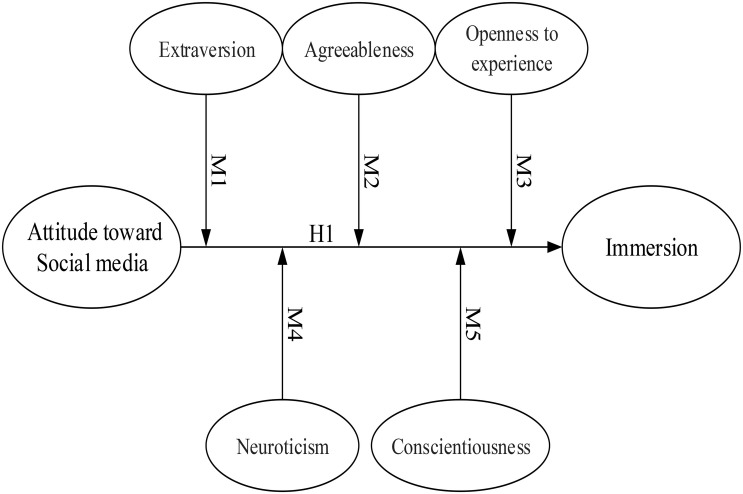
Research framework.

The primary purpose of the present research was to examine whether attitudes toward social media and the Big Five personality factors moderate and/or predict immersion. The hypothesized model was constructed using (1) the exogenous variable (attitude toward social media), (2) the endogenous variable (immersion), and (3) the moderator variables (the 5 personality traits of extraversion, agreeableness, openness to experience, neuroticism, and conscientiousness). The hypotheses are numbered and illustrated in the proposed path model, shown in Figure 1.

### Instruments

There were two self-report instruments that participants accessed and completed online: the “Attitudes, Immersion, and Personality Traits” (AIPT) questionnaire and the “Parent–Child Relationship” (PCR) inventory. The AIPT questionnaire included four sections: (1) basic demographics and Internet usage, (2) attitude toward social media, (3) immersion experiences, and (4) personality traits. Measure development followed MacKenzie et al. (2011) and standardization procedures followed those suggested by DeVellis (2003). On the AIPT, the 8 items on attitude toward social media and 6 immersion items were adapted from previous studies (Jennett et al., 2008; Wang et al., 2012; Christou, 2014; Grinberg et al., 2014; Balakrishnan and Gan, 2016). The personality trait items were developed by McCrae and Costa (2004), Clemens et al. (2015), Tang et al. (2016), Choi and Shin (2017), and Xiao and Mou (2019), and consisted of five dimensions and 22 items. The scale dimensions were extraversion (five items), agreeableness (four items), openness to experience (four items), neuroticism (five items), and conscientiousness (four items). Respondents used a Likert four-point scale (1 = strongly disagree, 4 = strongly agree) to respond to AIPT attitude, immersion, and personality sections. Items for each of these AIPT sections are shown in the Appendix.

The questions in the PCR inventory were written from a student’s perspective to evaluate stories participants had heard about parent–child relationship. A pilot test using the questionnaire was conducted with 1,086 senior high school students in central Taiwan to evaluate the revised questionnaire in terms of readability, ease of understanding, and formatting. Students who participated in the pilot test were excluded from the subsequent study. Further, a Cronbach’s alpha test was performed to test the reliability and internal consistency of each of the 36 measured attributes. The alpha coefficients for all of the 36 attributes ranged from 0.70 to 0.93, exceeding the minimum value of 0.6 that is widely used to indicate reliability Hair et al. (2010).

### Sample and Descriptive Statistics

A large sample was recruited in order to avoid common method variance and to increase the reliability of study findings. Survey data were collected from students attending 150 different high schools or vocational high schools in Taiwan. Using a sampling frame from a master list of Taiwanese high schools, a probability-proportionate-to-size sampling method was used to systematically draw a random sample of schools. Two or three classes were selected randomly from each of these 150 schools. An academic affairs staff member at each school was contacted and asked to help facilitate the work of the present study. A stratified purposive sampling method was then employed to select participants; information about the survey questionnaires was distributed either at school or, when schools were unwilling to distribute the survey, through the mail.

All respondents participated on a voluntary basis and were assured that their answers were anonymous or confidential, and they could refuse participation at any time without consequences. A total of 12,000 participants accessed the survey link: of these, 2,563 were eliminated due to incomplete or invalid answers. Completed questionnaires from the remaining 9,633 respondents were used in data analysis (valid response rate was 80.3%). Of these valid respondents, 4,702 were males and 4,931 females, and the average age was 16.33 years (*SD* = 0.94 years). Slightly over half (59.0%) of the participants reported having a “good” parent–child relationship and 92.7% reported “home” as the primary place of Internet usage. Table 1 shows the demographic and Internet usage characteristics of the sample.

**TABLE 1:** Profiles of participants (*N* = 9633).

Factor/Level	*N*	%	Factor/Level		*N*		%
*Gender*			*Parent–child relationships*				
Male	4702	48.8	Very good		3455		35.9
Female	4931	51.2	Good		5687		59.0
*Place of Internet usage*			Not good		491		5.1
Home	8934	92.7	*Purpose of Internet usage*				
School’s computer room	235	2.4	Online dating		2882		10.0
Internet cafe	234	2.4	Online games		5328		18.5
Library	29	0.3	Online shopping		2418		8.4
Other	201	2.1	Information search		6230		21.7
			Browsing social networking sites (such as: Facebook, Twitter, Google+, LinkedIn, Blogger etc.)		8932		31.1
			Other		2973		10.3
Item				Mean		S.D	
Age (years)				16.33		0.94	
Average weekly online leisure activities (time in minutes, non-vacation)				8.65		7.34	
Average weekly online leisure activities (time in minutes, vacation)				8.41		4.37	

### Statistical Analysis

Structural equation modeling is a widely accepted method used to gauge the validity of theories with empirical data and is used in comprehensive, combined analysis of both measurement models and structural models. One of the most common SEM techniques is partial least squares (PLS) (Chin, 1998). PLS, a component-based technique that uses a least-square estimation procedure, may be used for both construct validity and structural validity as well as to analyze measurements and structural models. This study used PLS with bootstrapping to test and validate the proposed model and the hypothesized relationships among the constructs.

Depictions of models that contain moderators that are obtained using PLS differ significantly from those that are obtained using traditional research model representations. In a PLS model, the moderators (personality traits in the current model) are shown as independent variables with a direct path to immersion. These interactive measures multiply every indicator in the moderator by every indicator in the independent variable, following Chin et al. (2003). Conceptually, the interaction construct (personality traits multiplied by attitude toward social media) is depicted as having a direct path to immersion. Additionally, the present research uses PLS to analyze the research model.

## Results of Research

### Measurement Model Evaluation

The measurement model assessed the convergent validity and the discriminant validity of each first-order construct. Each first-order construct was modeled as a reflective latent construct that accounted for its indicators. Three criteria were considered for assessing convergent validity (Fornell and Larcker, 1981; Hair et al., 2010; Bagozzi and Yi, 2012): (1) all-item loading (λ), (2) investigation of reliability coefficients (Cronbach’s alpha) and composite reliability coefficients (CR), and (3) average variance extracted (AVE).

Table 2 shows the indices of reliability and convergent validities for the AIPT questionnaire. The standardized item loadings ranged from 0.70 to 0.85; all items were larger than 0.70 and significant (*p* < 0.05 level) (Hair et al., 2010). Internal consistency was assessed using Cronbach’s alpha coefficient for each of the multi-item factors included in the model. Cronbach’s alpha coefficients ranged from 0.77 to 0.90, suggesting a high level of reliability. In addition, all constructs displayed a higher Cronbach’s alpha coefficient than the 0.70 benchmark suggested by Hair et al. (2010). Composite reliability is a set of latent construct indicators that are consistent in their measurements. These composite reliability coefficients ranged from 0.85 to 0.92, higher than the 0.6 benchmark suggested by Fornell and Larcker (1981).

**TABLE 2:** Validity and reliability.

Construct	Mean	*SD*	Cronbach’s alpha	CR	AVE	*R*^2^
Attitude toward Social media (ATSM)	2.56	0.63	0.90	0.92	0.59	
Extraversion (EXT)	3.07	0.59	0.86	0.90	0.64	
Agreeableness (AGR)	3.05	0.51	0.80	0.87	0.64	
Openness to experience (OPEN)	3.01	0.56	0.79	0.85	0.58	
Neuroticism (NEUR)	2.48	0.68	0.84	0.87	0.57	
Conscientiousness (CONS)	2.95	0.55	0.77	0.85	0.59	
Immersion (IMME)	1.69	0.63	0.82	0.87	0.54	0.44

Convergent validity was examined using AVE. Here, all constructs examined earned AVE values between 0.54 and 0.64, exceeding the minimum recommended value of 0.5 Fornell and Larcker (1981). Overall, the AVE from the constructs demonstrated satisfactory reliability and validity. In addition, discriminant validity refers to the degree of distinctive concept measurements. As shown in Table 3, the discriminate validity values for all constructs were greater than 1.0, indicating an appropriate level of discriminate validity (Fornell and Larcker, 1981; Hair et al., 2010, 2016). Overall, the constructs demonstrated satisfactory reliability, convergent validity, and discriminant validity, which justified proceeding to the next step of estimating the structural model.

**TABLE 3:** Correlation matrix and square root of the AVE.

	ATSM	EXT	AGR	OPEN	NEUR	CONS	IMME
ATSM	**0.59**						
EXT	0.14*	**0.64**					
AGR	0.13*	0.60*	**0.64**				
OPEN	0.18*	0.52*	0.54*	**0.58**			
NEUR	0.08*	−0.23*	−0.05*	−0.01	**0.57**		
CONS	0.07*	0.38*	0.44*	0.45*	0.08*	**0.59**	
IMME	0.64*	0.08*	0.08*	0.15*	0.15*	0.06*	**0.54**
Discriminant validity	1.45	1.87	1.78	2.02	11.16	2.88	1.33

*p < 0.05; Diagonal elements (in bold) are the square root of the average variance extracted (AVE). Convergent validity = AVE ≧ 0.5. Discriminant validity coefficient = AVE/(Correlation)^2^; where (Correlation)^2^ = highest (Correlation)^2^ between factor of interest and remaining factors.

### Hypothesis Testing

**FIGURE 2:**
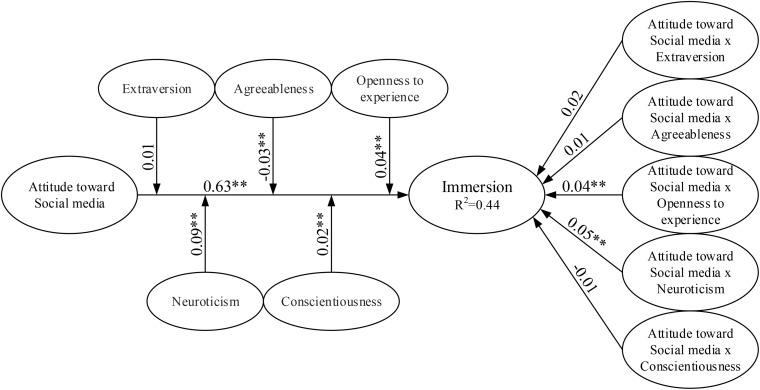
Results of the structural model testing. Value on path: standardized coefficients (β); *R*^2^: coefficient of determination; and ^∗^*p* < 0.1, ^∗∗^*p* < 0.05.

To test the research hypotheses, the paths between constructs were specified to build a structural model that matched the proposed relationships. Figure 2 shows the results of the SEM estimation, including standardized coefficients for each hypothesized path in the model, with significance based on one-tailed *t*-tests, and the amount of variance explained (*R*^2^). PLS analysis uses *R*^2^ values as a goodness-of-fit measure (Hulland, 1999). Table 4 reports the standardized beta-coefficients from the estimated structural model as well as the associated *t*-values for each construct.

**TABLE 4:** Estimation results for hypotheses.

Construct	Model 1		Model 2		Model 3	
	β	*t*-value	β	*t*-value	β	*t*-value
Attitude toward Social media → Immersion	0.65**	113.38	0.64**	101.55	0.63**	98.75
**Moderator effect**						
Extraversion → Immersion			0.01	1.02	0.01	0.82
Agreeableness → Immersion			−0.03**	2.53	−0.03**	2.69
Openness to experience→ Immersion			0.04**	3.53	0.04**	3.72
Neuroticism → Immersion			0.09**	9.71	0.09**	9.84
Conscientiousness → Immersion			0.02**	2.81	0.02**	2.69
**Interaction effect**						
Attitude toward Social media X Extraversion → Immersion					0.02	1.29
Attitude toward Social media X Agreeableness → Immersion					0.01	0.29
Attitude toward Social media X Openness to experience→ Immersion					0.04**	3.36
Attitude toward Social media X Neuroticism → Immersion					0.05**	4.54
Attitude toward Social media X Conscientiousness → Immersion					−0.01	0.88
Immersion (*R*^2^)	0.43		0.44		0.44	

*p < 0.1, **p < *0.05*.

Based on the moderator analysis method proposed by Baron and Kenny (1986), three models were explored. Model 1 explored the effects of the independent variable (attitude toward social media) on the dependent variable (immersion). Model 2 investigated the effects of the independent variable (attitude toward social media) and moderators (extraversion, agreeableness, openness to experience, neuroticism, and conscientiousness) on the dependent variable (immersion). Note that even when the independent variable and/or moderators are not significant independent predictors, they may interact. Model 3 investigated the interaction of attitude toward social media and each of the five personality traits on the dependent variable (immersion).

Among the Big-Five personality traits, openness to experience and neuroticism (hypothesis M3 and hypothesis M4) moderated the effects between attitude toward social media and immersion (M3: β = 0.04, *p* < 0.05; M4: β = 0.05, *p* < 0.05) and each interacted with attitude toward social media to positively affect immersion. That is, the effect of attitude toward social media on immersion increased as openness to experience and neuroticism increased. Thus, this study finds support for hypothesis M3 and hypothesis M4. This is similar with some previous studies (Kim et al., 2013; Chen et al., 2015; Xiao and Mou, 2019). In addition, the Big-Five personality traits, extraversion, agreeableness, and conscientiousness, did not have moderating effects on the relationship between attitude toward social media and immersion (M1: β = 0.02, M2: β = 0.01, and M5: β = −0.01, respectively, *p* > 0.05); thus, hypothesis M1, hypothesis M2, and hypothesis M5 were rejected. Finally, the construct of attitude toward social media had a significant positive effect on immersion (β = 0.63, *p* < 0.05). This implies that attitude toward social media is the determinant of immersion. Thus, this study finds support for hypotheses 1. In summary, the results showed that attitude toward social media had a significant influence on immersion and the interaction among attitude toward social media, openness to experience, and neuroticism has a positive influence on immersion, with the explained variance (*R*^2^) at 44.0%. Based on Cohen’s classification system (Cohen, 1988), the effect sizes for the association between variables were small, between 0.12 and 0.30 (mean 0.21). Figure 2 shows the full results of the moderation analysis, including the structural path estimates and explained variances.

## Discussion

This study investigated attitudes toward social media in terms of immersion in social media use for entertainment, which examined whether attitudes toward social media and the Big-Five personality factors moderated/predicted immersion. Questionnaire data from 9,633 students from senior high schools and vocational high schools in central Taiwan were collected and analyzed in order to test research hypotheses. There were several main findings.

First, there was a significant positive correlation between attitude toward social media and social media immersion. In Taiwan, information technology education has been implemented in elementary schools to enhance the ability of logic thinking and problem solving and national competitiveness. The school has taught the knowledge, classification, and use of social media. Through information technology education, young adults can learn about social media. When young adults’ increase their attitudes toward social media, they can then increase their immersion. Weibel et al. (2008) posited that presence is a subjective feeling when one is immersed in a virtual space. Therefore, this study considered that attitudes of high school students toward social media should be identified and guidelines should be developed to prepare students to effectively use leisure and entertainment platforms. In addition, when students were completely focused on social media, without any distractions, there was immersion.

Study participants perceived social media as a tool for social, leisure, and entertainment purposes as well as for educational purposes. They expressed positive attitudes and demonstrated positive outcomes related to social media usage, which supported the effectiveness of the government policy in Taiwan. When students had a positive attitude toward social media, they used social media more frequently to engage in related Internet leisure activities. During this engagement, young adults were immersed in social media, losing track of time and awareness of the real world. In addition, Jennett et al. (2008) found that the more immersed players were in a game, the longer they needed to readjust to the real world. That is, as information technology advances, social media may have a negative impact on students as they become immersed in virtual realities and disengage from the real world.

Further, participants were assessed in terms of the five Big-Five personality factors (extraversion, agreeableness, openness to experience, conscientiousness, and neuroticism) to provide empirical support for the structural model. The analysis demonstrated that interactive effects between attitude toward social media and two of the Big-Five personality traits (openness to experience and neuroticism) on immersion. Specifically, the influence of attitude toward social media on immersion was moderated by the extent of young adults’ openness to experience and neuroticism. This empirical result is similar with some previous studies (Kim et al., 2013; Chen et al., 2015; Xiao and Mou, 2019). That is, young adults with neurotic personalities and openness to experience personality are more likely to understand the strength of social media (e.g., improves work efficiency, beneficial for learning, maintaining interpersonal relationships, and contributing to society) and might cause them during this interaction with social media, absorbed in what they were doing and became immersed in this specific interaction. However, the non-significant interaction among attitude toward social media and extraversion, agreeableness, conscientiousness, which is similar with previous studies (Chen et al., 2015; Choi and Shin, 2017). Specifically, the influence of attitude toward social media on social media immersion was not moderated by the young adults’ personality traits (extraversion, agreeableness, and conscientiousness). Moreover, the consistency of the behavioral model and the latent variables exhibited strong convergent and discriminant validities, suggesting that our model effectively predicted social media immersion in young adults. There was also a significant interaction between attitude toward using social media and personality characteristics. Participants who held a relatively more positive attitude toward social media and personality traits (openness to experience and neuroticism) tended to experience higher immersion. When participants had a good attitude toward the use of social media and felt that using social media was advantageous, they used social media more frequently to engage in leisure activities. Specifically, we found that the effect of attitude toward social media on immersion increased as openness to experience and neuroticism increased.

## Limitations

The present study was affected by several potential limitations. First, personality traits were used as moderating effect without investigation excessive use of social media. Therefore, we recommend that the present experimental design be used in future research to investigate variances in attitudes toward online media and differences in immersion between excessive use of social media and non-excessive use of social media as a reference for secondary education teachers, students, parents, school officials, and relevant authorities. Second, this study focused on a single factor only (attitude toward social media). Future research should consider other factors (e.g., the theory of motivation and other psychological traits such as self-efficacy) that may explain young adults’ immersion in social media. Finally, this research has relied on self-reports of attitudes toward social media, and immersion, which may elicit misreporting to avoid judgment and cause common method variance.

## Data Availability Statement

The raw data supporting the conclusions of this article will be made available by the authors, without undue reservation.

## Ethics Statement

Ethical review and approval was not required for the study on human participants in accordance with the local legislation and institutional requirements. All of the subjects were informed about the research and all of the participants who were enrolled in the study provided informed consent.

## Author Contributions

T-KY: research conceptualization, obtaining funding, interpretation of the data, study supervision, writing-original draft. N-HL: data collection, concept and design, interpretation of the data, statistical analysis, and writing–original draft. C-MC: data collection, data curation and statistical analysis, interpretation of data, and writing–original draft. All the authors wrote the manuscript together and approved the final manuscript.

## Conflict of Interest

The authors declare that the research was conducted in the absence of any commercial or financial relationships that could be construed as a potential conflict of interest.

## Appendix

**TABLE A1:** Measurement items.

Constructs	Items	Sources
Attitude toward Social media	I feel that the use of social media is beneficial for learning.	Christou, 2014; Zhang et al., 2015; Balakrishnan and Gan, 2016
	Social media has made a positive contribution toward society.	
	Social media allows me to get work done faster.	
	I feel that social media has allowed me to keep in touch with many people.	
	I enjoy using social media for instant messaging or other types of real-time communication.	
	I enjoy using social media to pass time and/or to have fun.	
	I feel that social media improves my productivity.	
	I enjoy browsing (surfing) websites without any specific purpose.	
Extraversion	I am talkative.	McCrae and Costa, 2004; Clemens et al., 2015; Tang et al., 2016; Choi and Shin, 2017; Xiao and Mou, 2019
	I generate a lot of enthusiasm.	
	I am socially outgoing.	
	I have an assertive personality.	
	I am full of energy.	
Agreeableness	I am helpful and unselfish with others.	
	I like to cooperate with others.	
	I am considerate and kind to almost everyone.	
	I have a forgiving nature.	
Openness to Experience	I have an active imagination.	
	I am inventive.	
	I am original and come up with new ideas.	
	I like to reflect on and play with ideas.	
Neuroticism	I worry a lot.	
	I can be tense.	
	I get nervous easily.	
	I can be moody.	
	I am depressed or sad.	
Conscientiousness	I do things efficiently.	
	I do a thorough job.	
	I make plans and follow through with them.	
	I persevere until the task is finished.	
Immersion	I was able to block out most other distractions during this interaction with social media.	Jennett et al., 2008; Grinberg et al., 2014; Burns and Fairclough, 2015
	I was absorbed in what I was doing during this interaction with social media.	
	I got distracted very easily during this interaction with social media.	
	I became immersed in this specific interaction during this interaction with social media.	
	My attention did not get diverted during this interaction with social media.	
	Time seemed to fly during this interaction with social media.	
